# Understanding the relationships between health spending, treatable mortality and economic productivity in OECD countries

**DOI:** 10.3389/fpubh.2022.1036058

**Published:** 2022-12-21

**Authors:** Viera Ivankova, Beata Gavurova, Samer Khouri

**Affiliations:** ^1^Institute of Earth Resources, Faculty of Mining, Ecology, Process Control and Geotechnologies, Technical University of Košice, Košice, Slovakia; ^2^Center for Applied Economic Research, Faculty of Management and Economics, Tomas Bata University in Zlín, Zlín, Czechia

**Keywords:** treatable mortality, respiratory diseases, health spending, GDP, health systems, OECD, productive population

## Abstract

**Introduction:**

Population health is one of the highest priorities for countries, which can translate into increased economic prosperity. This encourages research on health in an economic context.

**Methods:**

The objective was to assess the relationships between health spending, treatable respiratory mortality, and gross domestic product (GDP) in countries of the Organization for Economic Co-operation and Development (OECD). The research was conducted with respect to health systems (tax-based, insurance-based) and gender differentiation of the productive population (aged 25–64 years). Descriptive analysis, regression analysis, and cluster analysis were used to achieve the main objective. The data covered the period from 1994 to 2016.

**Results:**

The results of the regression analysis revealed negative relationships between health spending and treatable respiratory mortality in countries with a tax-based health system for male and female working-age populations, as well as in countries with an insurance-based health system for male population. This means that higher health spending was associated with lower treatable respiratory mortality. Also, lower treatable mortality was associated with higher GDP, especially in the male productive population from countries with an insurance-based health system. In this study, countries with a tax-based health system were characterized by higher health spending, lower rates of treatable mortality from respiratory system diseases, and higher GDP compared to countries with an insurance-based health system. Males reported a higher mortality rate than females. Among the countries with a tax-based health system, the United Kingdom and Latvia showed less positive outcomes, while Italy and Iceland were the countries with the most positive outcomes. Among the countries with an insurance-based health system, Hungary and Slovakia reported poor outcomes, while France, Switzerland and Luxembourg were characterized by very positive outcomes. The United States showed a high mortality rate despite its high economic outcomes, i.e., health spending and GDP.

**Discussion:**

Health care financing in particular is one of the instruments of health policy. It seems that the leaders of countries should ensure a sufficient level of health financing, as higher health spending can contribute to lower mortality rates in a country. This may translate into higher productivity. Especially countries with underfunded health systems should increase their health spending.

## 1. Introduction

Population health is of great importance for the economic life of countries and is therefore constantly at the center of social, professional and political debates. Moreover, the coronavirus disease 2019 (COVID-19) pandemic reinforces this fact. Population health is in itself a great motivator for action to improve it, but the economic power of health is another driver for country leaders to make appropriate efforts. That is why public officials and experts strive every day to improve the health of a population, using a variety of tools to do so. Health spending is a common element of high-quality health care, adequate accessibility and efficient delivery of health services. It is health spending, that enables all these attributes of health systems to be achieved. In a health-economic context, health spending is considered growth-enhancing because it can increase both the quantity and productivity of labor by ensuring prolonged good health (1–3). All these facts encourage research on health in an economic context. If health systems are well designed, they should generate comparable results, and it is therefore possible to assess health indicators and confront them with economic indicators across countries (4, 5). One useful health indicator is treatable mortality, which reflects the effectiveness of health care and allows comparisons between countries and their applied health systems, suggesting the high economic potential of this indicator (6). Nevertheless, this indicator is still poorly understood and there is a clear need for research to examine the significant factors associated with treatable mortality in order to reduce the number of deaths and obtain potential health and economic benefits (7, 8).

The previous findings were the motivation for conducting this study, which focuses on examining the relationships between health spending, treatable mortality from respiratory system diseases among productive males and females, and gross domestic product (GDP) in countries of the Organization for Economic Co-operation and Development (OECD). This research was driven by the undeniable importance of the issue, as evidenced by the excessive health and economic losses worldwide. In addition, the current era is characterized by medical practices, knowledge and innovation which, if health systems and their financing are properly set up, should prevent premature deaths of productive people contributing to GDP. The COVID-19 pandemic highlighted these facts. This disease is mentioned several times in the study, as the research topic deals with treatable mortality from respiratory diseases. COVID-19 is an infectious disease that can lead to severe respiratory disease with the possibility of death. During the COVID-19 pandemic, many people died from treatable diseases, and at the same time, there was a disaster in health systems.

The issue of the relationship between health spending, treatable mortality, and economic productivity is still unclear, despite several studies that have been conducted. The originality of the paper lies in the fact that it examines all 37 OECD countries divided according to the applied health system. The presented research offers a comprehensive view of this issue in the OECD. In addition, COVID-19, which mainly affects the respiratory system, has increased the attention of researchers to this diagnosis group. This paper takes into account not only the applied health system but also gender differentiation and, in addition, focuses exclusively on treatable mortality from diseases of the respiratory system in the working-age population. To the best of our knowledge, such a perspective has not been used in any research.

## 2. Literature review

In general, treatable mortality is a health indicator that includes those causes of death that are expected to be averted through effective medical interventions in the form of appropriate treatment and secondary prevention (9). Such deaths can usually be averted by the age of 75 years. Evidence shows that treatable mortality contributes significantly to both overall and premature mortality despite its declining trend (8, 10). This was also confirmed by Nolte and McKee (11), who examined treatable mortality in 16 high-income countries and found that deaths from treatable diseases accounted for 24% of mortality in the population aged under 75 years. For these reasons, many studies have examined treatable mortality as a whole or within selected diseases (12–19). These studies confirm the fact that treatable mortality is widely recommended as an indicator of health systems performance over time, and, in combination with other indicators, is able to identify areas of the health system that need improvement. Comparative analyses using this indicator have the power to quantify differences in health system performance between geographical locations and to show whether these differences diminish over time (20).

Following the above-mentioned findings, it is possible to point out large differences in treatable mortality between countries, but also between their regional areas. This was confirmed by Weber and Clerc (10), who focused their research on the countries of the European Union. Similar findings were revealed by Jarčuška et al. (21), who found an explicit difference in treatable mortality rates between more developed countries of Western, Northern and Southern Europe and less developed countries of Central and Eastern Europe. Nolte and Mckee (16) highlighted differences in treatable mortality between the United States and three European countries (France, Germany, and the United Kingdom) and concluded that the United States has a relatively high treatable mortality rate and lags behind the other three countries in terms of a slower decline. Differences among 32 OECD countries were also confirmed by Gianino et al. (18). At this point, it should be noted that significant differences can be observed not only at the geographical level, but also at the societal level (13, 22, 23). Gender differences in avoidable mortality, which includes treatable mortality, were confirmed in several studies (24, 25), with the results showing that males face a higher risk of death from avoidable diseases compared to females (26, 27). All these findings underline the need to take into account the variety of countries with different health systems, as well as gender considerations, when examining treatable mortality.

It is understandable that the objective of any country's health system is to eliminate treatable mortality, as this effort can translate into health and economic gains (28, 29). In this context, Jarčuška et al. (21) found a strong negative relationship between treatable mortality and life expectancy at birth in the European Union. The findings of Verstraeten et al. (30) also indicated that reductions in treatable mortality may lead to gains in life expectancy. This can translate into longer working lives for individuals and higher productivity for countries, resulting in economic benefits. From the opposite perspective, it can be pointed out that poor health status in a country may be reflected in other socio-economic areas such as reduced employment, reduced productivity of a population, increased need for social protection, or reduced economic performance or competitiveness, and these are the attributes that underpin developed countries. As the productivity of small and medium-sized enterprises in particular depends on a healthy working-age population in a country, the economic importance of health is indisputable (31, 32). Alkire et al. (33) argued that the unjustifiability of treatable deaths brings economic losses in the form of a decline in the GDP of countries. The authors also revealed that treatable deaths can cause a cumulative loss of economic output of $11.2 trillion in low- and middle-income countries between 2015 and 2030. At the same time, it is possible to develop the idea that treatable mortality contributes significantly to overall mortality, and thus a reduction in treatable mortality could also be reflected in overall mortality. In this context, reductions in population mortality have been shown to improve the attributes of countries' economic life, not least GDP and economic development (34, 35). It is for these findings that special attention should also be paid to treatable mortality and its reduction. In other words, reductions in treatable mortality are desirable from both a health and an economic point of view. Based on the above, there is an assumption about the relationship between treatable mortality from various causes and GDP.

Health care financing, represented by health spending, appears to play an important role in the issue of reducing treatable mortality. This is supported by the findings from Heijink et al. (36), which showed a statistically significant negative relationship between health spending and avoidable mortality, which includes treatable mortality in addition to preventable mortality. According to the authors, most countries with above-average growth in health spending also show an above-average reduction in avoidable mortality. Currie et al. (37) took a different perspective on the issue and also highlighted that increasing health care financing for deprived areas can contribute to a significant reduction in treatable mortality inequalities and can thus help to converge health outcomes between rich and poor areas. In terms of mortality as such, it also appears that health spending, together with other social factors, can contribute to improving the health status of the population. Based on the above, there is an assumption about the relationship between health spending and treatable mortality from various causes.

Mortality from diseases of the respiratory system is undoubtedly considered a health burden. This is evidenced by the fact that respiratory diseases accounted for 7.5% of all deaths in the European Union in 2016 (38). These diseases are also one of the major causes of avoidable premature mortality (39), with asthma and chronic obstructive pulmonary disease (COPD) being a worldwide public health problem (40, 41). Thus, the impact of respiratory diseases is considerable, which is why international organizations call for increased attention to be paid to the respiratory health of populations as a priority in public health decision-making (42). To make matters worse, COVID-19, caused by the severe acute respiratory syndrome coronavirus 2 (SARS-CoV-2), has exacerbated already critical conditions (43). All of this can lead to huge economic losses, either in terms of significant costs for health services or in terms of lost production for businesses in general.

At this point, fragmented evidence should be pointed out. In terms of the economic nature of treatable mortality, the literature lacks a comprehensive view of respiratory disease mortality in the working-age population in OECD countries. Therefore, until this time, claims about relationships have been mere conjecture. Based on the current literature, the authors of this study set out to answer research questions focused on the diagnosis group of respiratory diseases. From the above-mentioned findings, it can be concluded that aspects such as health spending, GDP, as well as health systems may play an important role in the issue of treatable mortality. It is also necessary to distinguish between male and female mortality. On this basis, the following research has taken all these aspects into account when examining treatable mortality from respiratory system diseases in the productive population (aged 25–64 years).

## 3. Methodology

### 3.1. Research objective

The main objective was to assess the relationships between health spending (HS), treatable mortality from respiratory system diseases (RSP) and gross domestic product (GDP) in OECD countries. The research was conducted with respect to the health systems applied in the countries, as well as gender differentiation.

With respect to the main objective, the following research questions were formulated:

- RQ1: Are there statistically significant relationships between HS and male/female RSP in OECD countries with a tax-based health system?
- RQ2: Are there statistically significant relationships between HS and male/female RSP in OECD countries with an insurance-based health system?
- RQ3: Are there statistically significant relationships between male/female RSP and GDP in OECD countries with a tax-based health system?
- RQ4: Are there statistically significant relationships between male/female RSP and GDP in OECD countries with an insurance-based health system?

### 3.2. Research data

International databases, namely those of the OECD and WHO, provided economic and health data for research purposes. These data covered the period from 1994 to 2016, and were collected for all available years (i.e., for each year in which a country reported a value). The study period was chosen based on the availability of a data base sufficient for the analysis. As it was data on the specific number of deaths for specific treatable diagnoses of the respiratory system individually for working-age females and males, the published data were limited. This type of data is updated with little regularity. This was included among the limitations of the research.

The analyses included two economic variables obtained from the OECD database. First, health spending (HS) as a total share (%) of GDP capturing final consumption of health care goods and services, including personal health care (curative care, rehabilitative care, long-term care, ancillary services and medical goods) and collective services (prevention and public health services, health administration), but excluding investment spending (44). Second, gross domestic product (GDP) representing economic activity in terms of the added value generated by production in a country in a given year. This variable was expressed in US dollars per capita (economy-wide PPPs) (45).

The only health variable was treatable mortality from respiratory system diseases (RSP) collected from the WHO database (46). This group of diagnoses was selected because of its frequent occurrence in the population. The variable captured deaths from respiratory causes that can be avoided by early and effective health interventions, including secondary prevention and treatment (after the onset of respiratory diseases to reduce mortality) (9). According to the OECD and Eurostat list, the following diagnoses were included in the group of treatable respiratory causes of death, identified by the International Classification of Diseases (ICD-10) codes: J00–J12, J15–J20, J22–J47, J80–J81, J85–J90, J93–J94, J98 (9). These mortality data were provided separately for each age and gender category, and the research presented in this study was on the productive population aged 25–64 years. Taking into account gender differentiation, the collected mortality data were recalculated per 100,000 females aged 25–64 years as well as per 100,000 males aged 25–64 years. Data on population were obtained from the United Nations database as part of the “World Urbanization Prospects” report (47).

### 3.3. Research subjects

The research covered all 37 OECD countries. These countries were selected on the assumption that they have a well-developed health system that provides both primary and specialized (secondary and tertiary) health care. Their common feature is the overcoming of various challenges, which may include the economic nature of health as an output of the efficiency of health systems aimed at improving the health status of the population. Each of the countries provides health care to different degrees, which may be reflected in the health status of the population, but the essence of each OECD country is to ensure a better life for the population while improving the prospects for stronger and more equitable economies and societies.

OECD countries divided into two groups based on their applied health system:

- Countries with a tax-based health system: Australia (AU), Canada (CA), Denmark (DK), Finland (FI), Iceland (IS), Ireland (IE), Italy (IT), Latvia (LV), Norway (NO), New Zealand (NZ), Portugal (PT), Spain (ES), Sweden (SE), the United Kingdom (GB);
- Countries with an insurance-based health system: Austria (AT), Belgium (BE), Chile (CL), Colombia (CO), the Czech Republic (CZ), Estonia (EE), France (FR), Germany (DE), Greece (GR), Hungary (HU), Israel (IL), Japan (JP), Korea (KR), Lithuania (LT), Luxembourg (LU), Mexico (MX), the Netherlands (NL), Poland (PL), Slovakia (SK), Slovenia (SI), Switzerland (CH), Turkey (TR), the United States (US).

In 14 countries the health system was tax-based and in 23 countries the health system was insurance-based. Latvia changed its health system from insurance-based to tax-based in 2011; therefore, only the most recent data were included in the analyses.

The classification of OECD countries in terms of health systems was based on criteria from surveys of health system characteristics in OECD countries in 2008, 2009, 2012, and 2016 (48, 49), on criteria from the Country Health Profiles in 2017 and 2019 (50, 51), as well as on data provided on the websites of the ministries of health of each of the analyzed countries. These criteria are offered in the original surveys and reports.

### 3.4. Statistical analysis

In the first statistical step, a descriptive analysis offered a first look at the data using measures such as arithmetic mean, median, standard deviation, quartiles, minimum and maximum, skewness and kurtosis. In the second statistical step, the significance of the relationships between HS, RSP and GDP was assessed by a panel regression analysis, in which robust methods were used to estimate the coefficients (HC3). The study used robust panel regression models with fixed or random effects. The Arellano method (fixed effects models) and the White 2 method (random effects models) were used to assess the significance of the coefficients. The relationships were analyzed in four variants of the models as follows: “One-way (individual) effect Fixed effect model” (Arellano), “One-way (individual) effect Random effect model” (White 2), “Two-ways effects Within (fixed) effect model” (Arellano), “Two-ways effects Random effect model” (White 2). Prior to this analysis, panel diagnostics was performed to select an appropriate regression model. The *F*-test for the presence of individual effects (or time effects) and the Hausman test were chosen to test the assumptions. In the last third statistical step, a cluster analysis was used to provide a multivariate view of the relationships. The silhouette method (52) was chosen to estimate the optimal number of clusters, and the Partitioning Around Medoids (PAM) method and the Manhattan distance (53) were chosen to determine individual clusters. Prior to conducting this analysis, male/female mortality data (RSP) were averaged over the observed period for each country. The economic variables (HS, GDP) were also averaged, but gender differentiation was not applied. Subsequently, the averaged variables were standardized on a scale of 0–1, with 1 meaning the most positive score and 0 meaning the least positive score. After this step, the score of the economic variables were averaged again to form one economic score for each country. This helped to create two variables evaluating economic variables and a mortality variable against which countries were assessed in the cluster analysis. In this way, the cluster maps enabled the classification of countries.

The analytical processing was performed in the programming language R v 4.1.1 (RStudio, Inc., Boston, MA, USA).

## 4. Results

This section presents the main results of descriptive analysis, regression analysis, and cluster analysis. All these analyses respected the classification by health systems and gender.

Table 1 presents the results of the descriptive analysis of the analyzed economic and health indicators. With a focus on HS, it can be concluded that countries with a tax-based health system reported a higher level of spending on health care (Mean = 8.68; Median = 8.69) compared to countries with an insurance-based health system (Mean = 7.98; Median = 7.13). In a tax-based health system, a minimum level was 5.40%, and this was the case for Latvia in 2013. Among countries with an insurance-based system, Korea showed a minimum level of 3.35% in 1995. From the opposite perspective, Sweden, as a country with a tax-based health system, reported a maximum level of 10.98% in 2014. In an insurance-based health system, a maximum level was 16.71%, which was reported by the United States in 2015. In terms of economic productivity, countries with a tax-based health system showed a higher level of GDP (Mean = 35,565.12; Median = 34,198.62) than countries with an insurance-based health system (Mean = 28,591.40; Median = 26,585.63). Among countries with a tax-based health system, a minimum level of 19,887.83 USD per capita was identified for Latvia in 2011, while Norway reported a maximum level of 66,956.29 USD per capita in 2013. In an insurance-based health system, a minimum level of 6,554.63 USD per capita was identified for Colombia in 1999 and a maximum level of 103,787.97 USD per capita for Luxembourg in 2015.

**Table 1:** Descriptive statistics of economic indicators and treatable mortality from respiratory system diseases classified by gender and health system.

	**Tax-based health system**				**Insurance-based health system**			
	**HS**	**GDP**	**RSP-M**	**RSP-F**	**HS**	**GDP**	**RSP-M**	**RSP-F**
n	216	216	216	216	389	406	406	406
Miss	0	0	0	0	17	0	0	0
Mean	8.68	35,565.12	11.50	7.98	7.82	28,591.40	17.77	8.05
Median	8.69	34,198.62	9.64	7.13	7.15	26,585.63	16.23	7.67
St Dev	1.07	9,402.50	5.66	4.06	2.45	16,312.20	9.89	3.94
Skew	−0.25	0.80	2.28	1.05	1.16	1.51	1.33	0.99
Kurt	0.55	0.78	8.56	0.77	1.76	3.63	1.95	1.24
Min	5.40	19,887.83	3.49	2.35	3.35	6,554.63	2.76	1.82
Max	10.98	66,956.29	42.87	21.42	16.71	103,787.97	62.25	25.31
Q1	8.02	28,704.91	8.01	5.26	6.08	16,451.52	10.35	4.82
Q3	9.32	41,523.52	15.07	9.64	9.55	35,896.24	21.39	10.12

HS, health spending in % of GDP; GDP, gross domestic product per capita; RSP-M, treatable mortality from respiratory system diseases per 100,000 males aged 25–64 years; RSP-F, treatable mortality from respiratory system diseases per 100,000 females aged 25–64 years; n, number of observations; Miss, missing values; St Dev, standard deviation; Skew, skewness; Kurt, kurtosis; Min, minimum; Max, maximum; Q1, first quartile; Q3, third quartile.

Source own calculations based on available data (44–46).

Table 1 also shows descriptive statistics for RSP as an indicator of treatable mortality in the productive population. In this case, in addition to classification by the health system, classification by gender was also applied. A first look at the results revealed that countries with an insurance-based health system reported a higher mean mortality rate for both males and females (Mean: males = 17.77; females = 8.05) compared to countries with a tax-based health system (Mean: males = 11.5; females = 7.98). From a gender point of view, males showed higher mortality rates than females in both health systems. In other words, males were disadvantaged in terms of RSP. In a tax-based health system, males reported an average of 3.52 more deaths per year due to respiratory diseases compared to females. A maximum rate was 42.87 deaths per 100,000 males aged 25–64 years, which was observed in Latvia in 2013. On the other hand, Iceland in 2015 showed a minimum rate of 2.35 deaths per 100,000 females aged 25–64 years. Focusing on countries with an insurance-based health system, males reported an average of 9.72 more deaths per year due to respiratory diseases than females. This also indicates that countries with an insurance-based health system were characterized by a larger gender gap in terms of RSP. Lithuania was identified as a country with a maximum mortality rate of 62.25 deaths per 100,000 males aged 25–64 years in 2007, while Korea reported a minimum rate of 1.82 deaths per 100,000 females aged 25–64 years in 2009.

Based on the above-mentioned results, it was possible to assume that there was a relationship between economic and health indicators, as countries with a tax-based health system that showed higher levels of economic indicators (HS, GDP) also showed lower mortality rates (RSP). On the other hand, countries with an insurance-based health system showed lower levels of economic indicators, but also higher mortality rates. The following regression analysis focuses on examining the significance of the relationships between HS, RSP and GDP, respecting the classification by health system and gender. However, prior to conducting the regression analysis, assumption testing was performed to select an appropriate regression model. The results of the test statistics are shown in Table 2.

**Table 2:** Testing the assumptions for the selection of regression models.

		***F*-test—** **countries** **(*p*-value)**	***F*-test—** **years** **(*p*-value)**	**Hausman** **test** **(*p*-value)**	**Model**
**Tax-based health system**					
Male	HS → RSP	133.829 (< 0.001)	1.065 (0.388)	4.759 (0.029)	One-way fixed
	RSP → GDP	9.020 (< 0.001)	7.417 (< 0.001)	6.504 (0.011)	Two-ways fixed
Female	HS → RSP	129.632 (< 0.001)	1.311 (0.168)	8.578 (0.003)	One-way fixed
	RSP → GDP	13.963 (< 0.001)	5.960 (< 0.001)	9.357 (0.002)	Two-ways fixed
**Insurance-based health system**					
Male	HS → RSP	104.373 (< 0.001)	0.346 (0.998)	0.026 (0.871)	One-way random
	RSP → GDP	57.540 (< 0.001)	3.757 (< 0.001)	0.026 (0.871)	Two-ways random
Female	HS → RSP	101.935 (< 0.001)	0.344 (0.998)	0.229 (0.632)	One-way random
	RSP → GDP	64.463 (< 0.001)	3.955 (< 0.001)	0.259 (0.611)	Two-ways random

HS, health spending in % of GDP; RSP, treatable mortality from respiratory system diseases per 100,000 males/females aged 25–64 years; GDP, gross domestic product per capita.

Source own calculations based on available data (44–46).

Based on the results in Table 2, it can be stated that a one-way fixed effects model was preferred to assess the relationship between HS and RSP in a tax-based health system, as evidenced by the results of the *F*-tests indicating statistically significant effects only in the country structure and the results of the Hausman test indicating the choice of a fixed effects model (*p*-value < 0.05). In contrast, a one-way random effects model was recommended to assess the relationship between HS and RSP in an insurance-based health system, with the results of the *F*-tests showing statistically significant effects only in the country structure and the results of the Hausman test favoring the choice of a random effects model (*p*-value > 0.05).

When focusing on the relationships between RSP and GDP in a tax-based health system, the preference inclined toward a two-ways fixed effects model, as these cases showed statistically significant effects in the data structure for both countries and years, and their results of the Hausman test recommended a preference for a fixed effects model (*p*-value < 0.05). On the other hand, a two-ways random effects model was preferred to assess the relationships between RSP and GDP in an insurance-based health system, as the *F*-tests revealed statistically significant effects in the data structure for both countries and years, and the results of the Hausman test supported the choice of a random effects model (*p*-value > 0.05).

The following Table 3 provided the main results of panel regression models with fixed or random effects evaluating the significance of the relationships. It should be noted at this point that the authors' first intention was to show all of the original results of the panel regression models used, but in their interpretations the authors only considered the results of the recommended model, which was appropriate based on the testing of assumptions for the selection of the regression models. Their results were accepted.

**Table 3:** Panel regression analysis—relationships between economic and treatable mortality indicators classified by gender and health system.

			**One-way** **random**	**One-way** **fixed**	**Two-ways** **random**	**Two-ways** **fixed**
**Tax-based health system**						
Male	HS → RSP	*R* ^2^	0.166	0.122	0.120	0.029
		α	19.67^***^		19.65^**^	
		β	−0.81^***^	**[**–0.76^*^]	−0.81	−0.55
	RSP → GDP	*R* ^2^	0.096	0.084	0.142	0.064
		α	44707.60^***^		30632.46^***^	
		β	−789.75^***^	−1148.59^**^	80.08	**[362.37]**
Female	HS → RSP	*R* ^2^	0.129	0.132	0.001	0.037
		α	13.14^***^		12.70^***^	
		β	−0.61^***^	**[**–0.62^*^]	−0.53^*^	−0.43^**^
	RSP → GDP	*R* ^2^	0.085	0.118	0.008	0.021
		α	44408.84^***^		30925.14^***^	
		β	−1213.82^***^	−1747.85^**^	100.05	**[298.54]**
**Insurance-based health system**						
Male	HS → RSP	*R* ^2^	0.032	0.026	0.082	0.001
		α	23.07^***^		22.18^***^	
		β	**[**–0.75^***^]	−0.72	−0.63^***^	0.14
	RSP → GDP	*R* ^2^	0.110	0.099	0.198	0.026
		α	39654.84^***^		30004.27^***^	
		β	−633.19^***^	−622.65^*^	**[**–176.19^**^]	−147.37
Female	HS → RSP	*R* ^2^	0.015	0.011	0.035	0.001
		α	6.27^***^		6.50^***^	
		β	**[0.20** ^*^ **]**	0.19	0.17	0.02
	RSP → GDP	*R* ^2^	0.005	0.001	0.028	0.001
		α	28548.15^***^		27040.36^***^	
		β	7.24	63.79	**[**–**28.50]**	−14.18

Sig.: ^***^p-value < 0.001, ^**^p-value < 0.01, ^*^p-value < 0.05, · *p*-value < 0.1.

HS, health spending in % of GDP; RSP, treatable mortality from respiratory system diseases per 100,000 males/females aged 25–64 years; GDP, gross domestic product per capita.

The accepted results are highlighted in bold and brackets [].

Source own calculations based on available data (44–46).

In each analyzed case, the balance statistics of the panel regression models argued in favor of considering a balanced model. This was supported by the acquired values of gamma (γ) and nu (ν). The closer their value was to 1, the more balanced the panel seemed. In terms of countries with a tax-based health system, the values were γ = 0.8463299 and ν = 0.9060825 when evaluating all the relationships. In terms of countries with an insurance-based health system, the values were γ = 0.7470208 and ν = 0.9352060 when evaluating the relationships between HS and RSP, and γ = 0.7376557 and ν = 0.9380606 when evaluating the relationships between RSP and GDP. The coefficients of determination (R2) were informative only and did not need to be considered in terms of model strength. The low value was due to the relatively low number of observations as a result of the classification structure of the panel data.

The results of the regression models provided in Table 3 revealed several significant relationships between the analyzed variables. Focusing on the recommended models in assessing the relationships, it can be concluded that HS was associated with RSP in both health systems and both gender categories.

Statistical significance at the level of α < 0.05 and negative coefficient estimates can be observed in countries with a tax-based health system in the population of males and females of productive age. This indicates that in these countries higher HS was associated with lower RSP, and vice versa. On this basis, it can be expected that in a country with a tax-based health system, the number of treatable deaths from respiratory system diseases in the productive population would decrease with an increase in HS.

In countries with an insurance-based health system, the situation was different. In the population of productive males, a significance of the relationship between HS and RSP was confirmed at the level of α < 0.001, and the coefficient estimate was again negative. In the population of productive females, statistical significance was confirmed at the level of α < 0.05, but in this case the coefficient estimate was positive. As mentioned above, a negative coefficient indicates that higher HS was associated with lower RSP, and vice versa. Conversely, a positive coefficient is indicative of the fact that in countries with an insurance-based health system, lower HS was observed along with lower RSP in the female productive population, and vice versa.

Either way, HS played an important role in RSP, especially for the productive population in countries with a tax-based health system and the population of productive males in countries with an insurance-based health system.

A significant relationship between RSP and GDP was confirmed only for productive males in countries with an insurance-based health system, with statistical significance at the level of α < 0.01. In this case, RSP was negatively associated with GDP. This means that fewer treatable deaths from respiratory system diseases among productive males were associated with higher GDP, and vice versa. This was true in countries with an insurance-based health system.

Looking at the results, it is possible to speculate that time explains changes in countries' economic productivity (GDP) to a greater extent than RSP. It can be assumed that the treatment of respiratory diseases requires high spending, thus draining resources in the economy, and this burden may also be reflected in the relationship between RSP and GDP.

The following cluster analysis was conducted to point to homogeneous groups of countries based on their economic (HS, GDP) and health (RSP) outcomes. In addition, this made it possible to assess each country in comparison with other countries applying the same health system. This health-economic assessment also respected gender differentiation, as males were more vulnerable in terms of mortality. It should be noted that the change in a country's position was driven by mortality outcomes separately for males and females, as the economic outcomes were the same for the entire population of a country. The upper right quadrant represents the most positive position, while the lower left quadrant represents the least positive position in terms of the assessment of countries.

**Figure 1:**
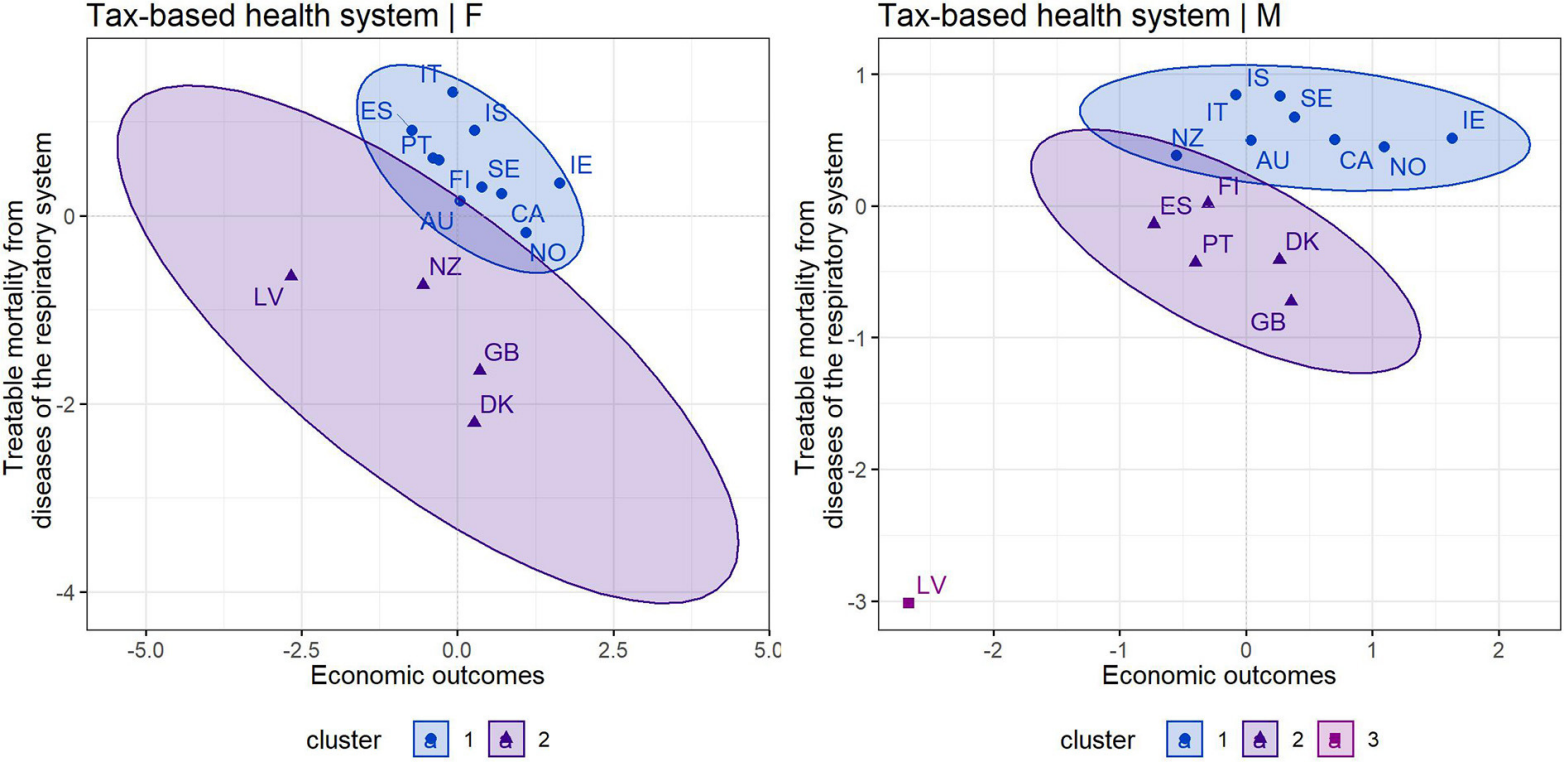
Cluster maps of countries applying a tax-based health system [left—female population, right—male population) [Source own processing based on available data (44–46)].

With a focus on the cluster maps presented in Figure 1, it can be stated that two clusters were recommended by the silhouette method to assess countries with a tax-based health system based on their economic outcomes (HS, GDP) and mortality outcomes (RSP) in the female population, and three clusters in the male population. Regarding the left cluster map, which took into account economic outcomes and female mortality, countries such as Denmark, the United Kingdom, Latvia and New Zealand were included in the second cluster with less positive assessment positions. On the other hand, Italy, Iceland, but also other countries were the countries in the first cluster that showed the most positive positions.

Focusing on the right cluster map, which covered the male population, only Latvia was in the third cluster. This country showed the least positive economic and mortality outcomes. The second cluster included the United Kingdom, Denmark, Portugal, Finland and Spain. The most positive positions were represented by the countries in the first cluster, namely Iceland, Italy, Sweden, Canada, Norway, Ireland, but also New Zealand and Australia. At this point, several findings should be emphasized. In addition to the least positively assessed Latvia, developed countries such as Denmark and the United Kingdom also showed less positive outcomes, especially in terms of mortality outcomes in both male and female populations. Interestingly, while Spain, Portugal and Finland were among the more positive countries in terms of female mortality, they were among the poorer countries in terms of male mortality. The opposite situation was observed in New Zealand. Overall, countries such as Ireland, Norway, Canada, Sweden, Iceland and Italy were very positive from a health-economic perspective.

**Figure 2:**
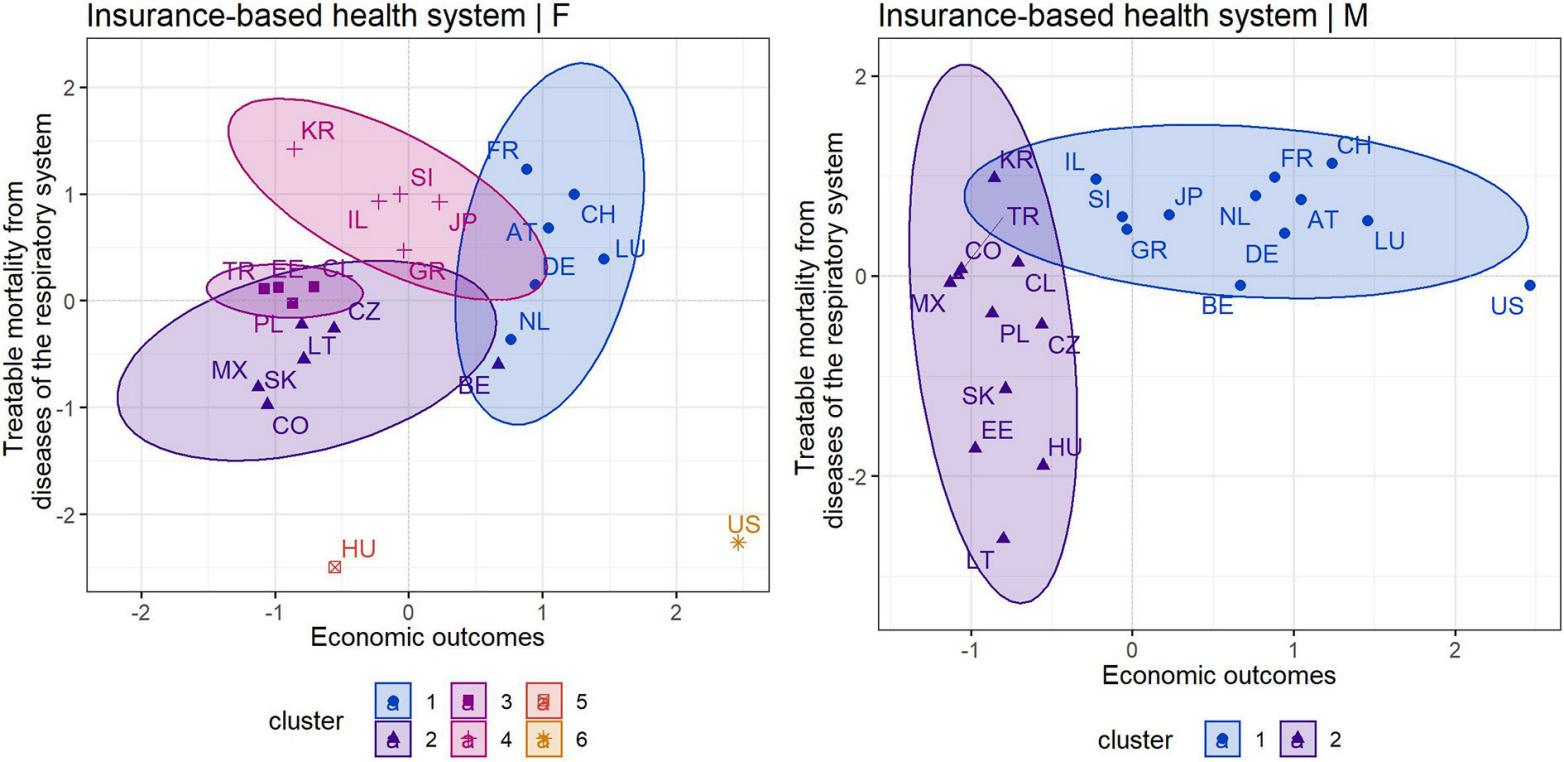
Cluster maps of countries applying an insurance-based health system [left—female population, right—male population) [Source own processing based on available data (44–46)].

Figure 2 shows the cluster maps for countries that apply an insurance-based health system. It can be noted that six clusters were identified for the assessment of economic outcomes (HS, GDP) and female mortality outcomes (RSP), while two clusters were identified for the assessment of economic outcomes and male mortality outcomes. With a focus on the left cluster map, which took into account the female population, attention should be paid to the United States (cluster 6), which reported a high mortality rate despite high economic outcomes, i.e., HS and GDP. This cannot be considered positive. Hungary (cluster 5) also showed a very poor position, indicating lower economic outcomes as well as a high mortality rate. Other less positive countries with an insurance-based health system were Colombia, Mexico or Slovakia. On the other hand, countries such as France, Switzerland, Luxembourg and Austria were among the countries with the most positive assessment positions.

Looking at the right cluster map covering the male population, countries such as Lithuania, Hungary, Estonia and Slovakia from the second cluster had the least positive positions, i.e., they showed the least positive economic and mortality outcomes. The first cluster included the countries with the most positive outcomes, in particular France, Switzerland, Luxembourg, Austria, but also others. The United States was considered an outlying country. In this case, given the high economic outcomes, a more positive mortality rate was expected.

## 5. Discussion

It goes without saying that developed countries consider the health of their populations to be one of their highest priorities and adjust their financing accordingly. It is clear from public databases that health spending has been growing in most OECD countries and, moreover, some countries have shown that their health spending has consistently grown faster than the rate of GDP growth (44). This study revealed that countries with a tax-based health system reported higher health spending than countries with an insurance-based health system. At the same time, countries with a tax-based health system showed lower rates of treatable mortality from respiratory system diseases compared to countries with an insurance-based health system. Finally, in terms of economic productivity, countries with a tax-based health system showed higher GDP than countries with an insurance-based health system. All of this contributes to understanding the economic perspective of treatable mortality (6). The findings also point to the wellknown fact that males face a higher risk of death from avoidable diseases compared to females (26, 27). In general, even Kim (54) agreed that the survival probability of males is disadvantaged compared to the survival probability of females. According to this author, socioecological factors play an important role in the issue of life expectancy and humans' survival probability of becoming centenarians. The different habits and health behaviors of males should also be emphasized. In this way, they are characterized by delaying seeking health care, non-adherence to treatment, premature discontinuation of treatment, as well as ignoring and downplaying health problems (55). Thus, the gender aspect should not be overlooked when designing effective interventions aimed at reducing treatable mortality in the productive population.

The study demonstrated that health spending is one of the important factors that should be considered when addressing treatable mortality from respiratory system diseases in the productive population. Comparable negative relationships were observed in countries with a tax-based health system for male and female populations, as well as in countries with an insurance-based health system for male population. This means that higher health spending was associated with lower treatable mortality. However, a positive relationship was found in countries with an insurance-based health system for the female population. In any case, diseases and acute inflammations of the upper and lower respiratory tract or pneumonia are widespread in the whole population; therefore, special attention should be paid to this group of diagnoses. The year 2020, in which the COVID-19 pandemic was in full force globally, is the year when it became clear that these diseases with viral infections can be very critical to health. At the same time, the pandemic showed the important role of health spending, as it made it possible to provide the necessary health care at a time when it was more than needed (56, 57). The results of the presented study also contribute to the understanding of this issue. In general, it can be agreed that health spending can help to reduce mortality and increase life expectancy, which is consistent with other studies (58–61). According to Makuta and O'Hare (62), public spending on health has a significant impact on health outcomes. Thus, public spending on health improves health outcomes. The findings of Budhdeo et al. (63) showed that a 1% decrease in health spending, measured as a share of GDP, was associated with a significant increase in all mortality metrics. This was confirmed in the short and long term. Therefore, policy measures taken in response to the pandemic should focus on health care financing in order to avoid a deterioration in the health status of the population. Lest it be forgotten, there are various health care resources, including health care facilities, a number of health professions, vaccinations, which may play an important role along with health spending (64).

In fact, every country should strive to reduce treatable mortality using all available tools, including health spending. These efforts lead to other beneficial consequences. According to Kiadaliri (65), reductions in avoidable (treatable and preventable) mortality can be clearly reflected in life expectancy. This may also bring economic benefits such as longer productive life and increased productivity of the population. The importance of this is underlined by the statements of international organizations that healthy populations are drivers of economic life, whether in terms of their higher productivity, longer working lives, or reduced burden of health and social spending (66, 67). Last but not least, health is a source of comparative economic development of countries (68). In the light of the results of this study, lower treatable mortality from respiratory system diseases was associated with higher GDP, especially in the male productive population from countries with an insurance-based health system. This is consistent with the findings of Fantini et al. (69), who also found a negative relationship between mortality and GDP per capita. In addition, Alkire et al. (33) emphasized that economic losses, such as a decrease in GDP, can be expected due to treatable mortality. In other words, reductions in mortality can be reflected in economic benefits (34). All of these findings suggest that public policy-makers need to recognize that if premature and treatable deaths did not occur, productive populations could continue to generate economic gains (35).

Population health should be a central social, professional and political issue, and general efforts should focus on improving it, which can bring economic additional benefits. Treatable mortality indicates the extent of a health system's contribution to population health, and it is important to look at this indicator when assessing health systems that vary by funding model (6). This study showed that countries within a single health system can be further sorted into several clusters, distinguishing between countries with less positive outcomes and countries with more positive outcomes. In this context, the United Kingdom and Latvia were among the countries with a tax-based health system that showed less positive outcomes. On the other hand, Italy and Iceland were the countries with the most positive outcomes. Among the countries with an insurance-based health system, Hungary and Slovakia had poor outcomes, while France, Switzerland and Luxembourg were characterized by very positive outcomes. The United States showed a high mortality rate despite its high economic outcomes, i.e., high health spending and GDP. The results clearly showed that countries vary from each other despite implementing the same health system, and they can be compared with other studies that have examined a similar problem (16, 18, 21, 70).

The difficult current period requires active efforts to improve the health status of the productive population, including a reduction in treatable mortality. To achieve this, the development and implementation of successful health policies and continuous improvement of health systems with sustainable and adequate health care financing are essential.

### 5.1. Implications for public policies

It is desirable for policy makers to use evidence on the relationship between population health and the economic life of countries. The presented study offers this evidence and supports the strengthening of health systems, which can translate into better population health and increased economic prosperity. This is both the objective and the challenge of every public health system. Health spending appears to be an aspect that can contribute to some extent to good health translating into the productivity of the economy. With a focus on the research presented in this study, treatable mortality is an important health indicator on which many surveys comparing countries and their health systems are based. The study contributes to the formulation of health policies and provides a supportive approach to the development of strategic plans with respect to particular diagnosis groups and gender specificities. Health care financing in particular is one of the instruments of health policy, and policy makers are in a unique position to adopt and promote evidence-based measures in an effort to improve population health, increase health system efficiency, as well as enhance economic productivity, which is linked to the competitiveness of countries in international comparisons. It seems that the leaders of countries should ensure a sufficient level of health financing, as higher health spending can contribute to lower mortality rates in a country. This may translate into higher productivity. Especially, countries with underfunded health systems should increase their health spending.

### 5.2. Limitations

Limitations of the study include the fact that it focuses only on OECD countries with two models of health systems and therefore its results cannot be generalized to other less developed countries or countries with different health systems. The unbalanced structure of the panel data can also be considered a limitation. This may have been due to the specific focus on a particular group of diagnoses, with not all countries publishing their health outcomes in detail. On the other hand, the study includes all available country-reported data. Regarding the limitations of the models, causality was not examined; therefore, the relationships revealed in the study cannot be seen as causal. All results can only be considered in terms of associations, and reasoning about causal relationships can be misleading. As it was data on the specific number of deaths for specific treatable diagnoses of the respiratory system individually for working-age females and males, the published data were limited. This type of data is updated with little regularity. As this was an overall view of the situation in the OECD across several classifications, it was not possible to filter the data further. Another limitation is that only health spending was considered. It is necessary to recognize that health spending is not the only important factor of treatable mortality that is related to the health system. There are many factors that the study did not account for. Future research should focus on other factors such as quality and accessibility of health care, qualifications of doctors, working conditions, equipment of health facilities, availability of medicines, and management of health facilities. At the same time, other socio-economic and environmental determinants of health should also be considered.

## 6. Conclusion

Deaths from respiratory system diseases that can be averted through health care need increased attention, especially in the context of the COVID-19 pandemic. This study emphasizes an economic perspective of this problem. The main objective was to assess the relationships between health spending, treatable mortality from respiratory system diseases and GDP in OECD countries, with the productive population as the focus of the research. The research was carried out taking into account the health systems implemented in these countries as well as gender differentiation. The main finding highlighted the important role of health spending in treatable mortality in countries with both tax- and insurance-based health systems. In this way, higher health spending can be expected to lead to lower treatable mortality from respiratory system diseases. Lower treatable mortality was associated with higher economic prosperity (GDP), especially in the male productive population from countries with an insurance-based health system. On this basis, the leaders of countries should ensure a sufficient level of health financing. Higher spending on health could help countries from both a health and economic point of view, and this should not be forgotten in the creation of public policies. In particular, countries with underfunded health systems should increase their health spending. In this study, countries with a tax-based health system were characterized by higher health spending, lower rates of treatable mortality from respiratory system diseases, and higher GDP compared to countries with an insurance-based health system. The results of the study provide a closer look at the health systems applied in OECD countries. In this context, the consideration of health systems is undoubtedly beneficial for future research efforts.

## Data availability statement

Publicly available datasets were analyzed in this study. This data can be found here: https://data.oecd.org/healthres/health-spending.htm#indicator-chart, https://data.oecd.org/gdp/gross-domestic-product-gdp.htm, https://www.who.int/data/data-collection-tools/who-mortality-database?fbclid=IwAR2gVBBqbMEUf6Y1g505FjN_hg77TkINf_VWGO3-efYrTr-J9sC7_Wkpy7Q, https://www.oecd.org/health/health-systems/Avoidable-mortality-2019-Joint-OECD-Eurostat-List-preventable-treatable-causes-of-death.pdf, https://population.un.org/wup/.

## Ethics statement

Ethical approval was not required for the study on human participants in accordance with the local legislation and institutional requirements. Written informed consent for participation was not required for this study in accordance with the national legislation and the institutional requirements.

## Author contributions

VI: conceptualization, methodology, formal analysis, investigation, resources, writing—original draft preparation, visualization, writing—review and editing, and supervision. BG: conceptualization, visualization, writing—original draft preparation, writing—review and editing, supervision, project administration, and funding acquisitions. SK: data curation, writing—original draft preparation, visualization, writing—review and editing, supervision, and funding acquisitions. All authors contributed to manuscript revision, read, and approved the submitted version.
